# Zika NS2B Protein: In Vitro Formation of Large Multimeric Networks

**DOI:** 10.3390/ijms27031504

**Published:** 2026-02-03

**Authors:** Caleb Ponniah, Wahyu Surya, Jaume Torres

**Affiliations:** School of Biological Sciences, Nanyang Technological University, 60 Nanyang Drive, Singapore 637551, Singapore; caleb001@e.ntu.edu.sg (C.P.); wsurya@ntu.edu.sg (W.S.)

**Keywords:** *Flaviviridae* family, Zika virus, multimerization, flavivirus, non-structural 2B protein, membrane proteins

## Abstract

Flaviviruses are responsible for significant morbidity and mortality worldwide. Despite intensive research, the structure and oligomerization properties of non-structural (NS) proteins, like NS2 or NS4, are still uncertain because of their high hydrophobicity. Solution NMR has shown that NS2B protein has two hydrophobic domains, organized as two short α-helical hairpins that contribute to both viral RNA replication and particle formation. These are separated by a hydrophilic loop that is a cofactor of protease NS3. However, the oligomerization behavior of NS2B has not been explored in detail. Herein, we have expressed Zika virus NS2B protein (ZIKV NS2B) and characterized its oligomerization in both detergent and lipids using crosslinking in liposomes, and mass photometry and analytical ultracentrifugation in detergent. We show that, in contrast to the small oligomers proposed earlier, ZIKV NS2B protein has a very complex oligomerization behavior, forming from dimers to very large multimers (>10) in both detergent and lipids. Although AlphaFold (AF) provided a model for monomeric NS2B that is consistent with available experimental data, no oligomeric model was predicted with confidence. We suggest that the role of the two short α-helical hairpins in membrane destabilization and reshaping host ER during viral infection may be aided or triggered by multimerization. Finally, although our results report a high tendency of NS2B to oligomerize, in the context of the infected cell, a biologically relevant multimeric complex may necessitate other viral proteins like NS4A or NS4B and/or host proteins.

## 1. Introduction

Viruses of the *Flaviviridae* family are responsible for significant morbidity and mortality worldwide but there is no efficient chemotherapy or vaccine available [1,2,3]. Despite efforts in the last few decades, our understanding of the protein roles in these viruses is in some cases incomplete, especially for membrane proteins that do not have an enzymatic or structural role, such as non-structural (NS) proteins NS2 or NS4, where in some cases even the number of transmembrane domains (TMDs) is still debated [4,5,6,7,8].

The genome of flaviviruses is a single-stranded positive-sense RNA molecule that is translated by the host cell machinery, producing a polyprotein that is cleaved by both viral and cellular enzymes. Structural proteins like capsid (C) bind RNA and trigger viral envelope formation and budding into endoplasmic reticulum (ER)-derived membranes. These membranes contain envelope (E) glycoprotein that forms a highly ordered array on the surface of the mature virion with premembrane/membrane prM [9,10], which protects the fusogenic E protein during transit from the ER lumen to the cell surface [11].

NS proteins are crucial in immune system evasion and in the formation of the replication complex (RC) [12,13,14], a virus-induced membrane network derived from the endoplasmic reticulum (ER) [15,16,17] where viral RNA is synthesized [18,19,20]. In flaviviruses, NS2 protein is split into NS2A and NS2B, whereas NS3 has protease (N-terminal) domain which requires the cytoplasmic loop of NS2B as a cofactor [21].

Flaviviruses include vector-borne human disease agents such as yellow fever virus (YFV), Zika virus (ZIKV), Dengue virus (DENV) and West Nile virus (WNV) [22,23,24]. In addition to C, E and prM, they encode another seven NS proteins (NS1, NS2A, NS2B, NS3, NS4A, NS4B and NS5), four of which are very hydrophobic and poorly characterized (NS2A, NS2B, NS4A and NS4B) [14]. Solution NMR experiments in detergent micelles have resulted in a model for NS2B where the two hydrophobic regions are in fact short transmembrane α-helical hairpins, as shown for DENV serotype 4 (DENV4) NS2B and ZIKV NS2B in LMPG or SDS micelles, respectively [25,26].

**Figure 1:**
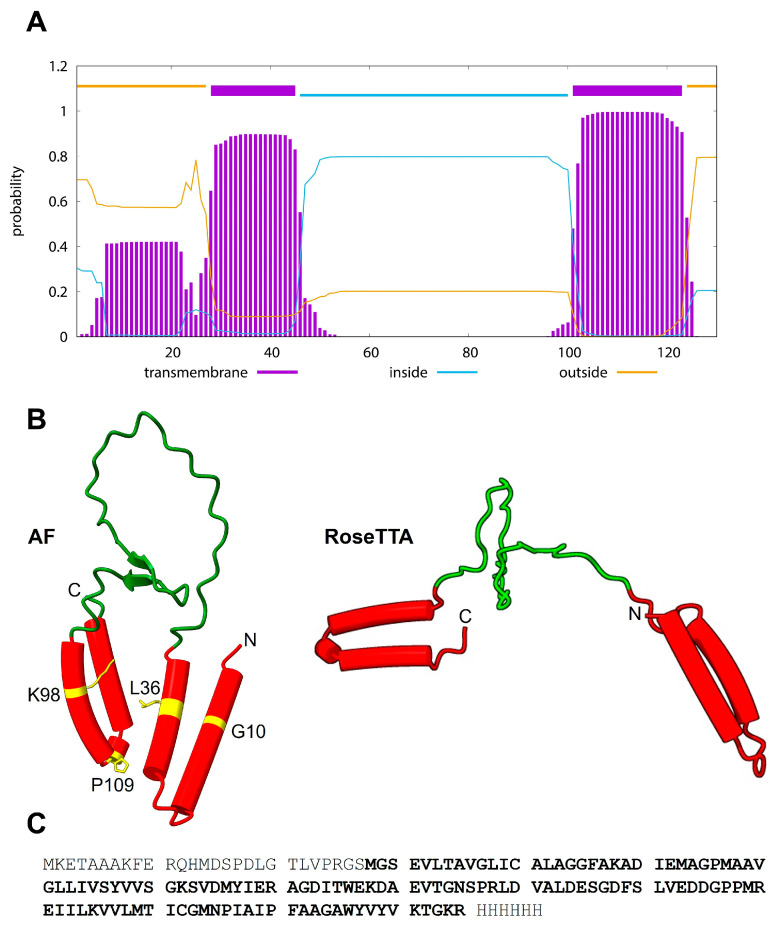
**Predicted structure of ZIKV NS2B monomer.** (**A**) TMHMM 2.0 prediction of TM domains; (**B**) structures predicted by AlphaFold and RoseTTAFold (https://robetta.bakerlab.org). Residues corresponding to mutations in JEV that severely attenuated or inhibited RNA synthesis [29] are shown in yellow, whereas red and green show helical and non-helical stretches, respectively; (**C**) ZIKV NS2B construct sequence used in this work consists of the full-length ZIKV NS2B protein (bold), an N-terminal S-tag and thrombin cleavage site, and a C-terminal 6-His tag. The MW of the construct is 17.3 kDa.

These two hydrophobic regions are predicted by TMHMM [27,28] (Figure 1A), whereas both AlphaFold (AF) and RoseTTa predict the two short α-helical hairpins (Figure 1B).

In Japanese encephalitis virus (JEV), the TM domains of NS2B were shown to contribute to both viral RNA replication and particle formation [29], and mutations in conserved residues within the hydrophobic regions severely attenuated or inhibited RNA synthesis while not affecting NS2B-NS3 protease activity. The corresponding residues mutated in ZIKV NS2B are shown in Figure 1B (left panel).

The membrane topology of NS2B was first obtained in DENV 4 [30,31] using NMR spectroscopy in detergent micelles. Four short membrane-buried α-helices (α1 to α4) were identified, consistent with two short α-helical hairpins [31], encompassing residues 4–19, 25–41, 90–105 and 112 to 125, respectively, whereas the intervening loop had a tendency to form β-strands, consistent with the β-strand structure observed when it forms a complex with the N-terminal protease domain of NS3 [32,33,34,35]. When not forming a complex with NS3, this cofactor region is highly dynamic [25,31,36,37,38]. It was suggested that the presence of small-XXX-small motifs might be important for TM–TM interactions. These hydrophobic domains are crucial for the attachment of NS3 to the membrane [29,39]. Conformational heterogeneity and oligomerization of NS2B were suggested from the broadened peaks in NMR HSQC experiments (DENV4 NS2B) [31] and from the presence of extra resonances (ZIKV NS2B) [25]. The latter study found that NS2B could form at least dimers and trimers when crosslinked with glutaraldehide (GA), whereas the construct NS2B-NS3 was reported to form trimers [40]. In DENV, NS2B can form a complex with NS3, NS4A and NS4B [41,42], although direct interaction between NS2B and NS4A or NS4B in any flavivirus has not been demonstrated. Recent AF3 predictions using complete flavivirus polyproteins [43] also suggest this possibility. Thus, oligomeric forms of NS2B might be important for viral replication, particle formation and replication complex formation [29,44] via formation of hetero-oligomers by interacting with NS2A, NS4A or NS4B [7]. Herein, we have explored the oligomerization behavior of ZIKV NS2B using both biochemical and biophysical techniques.

## 2. Results

### 2.1. Extraction and Ni-NTA Affinity Purification

**Figure 2:**
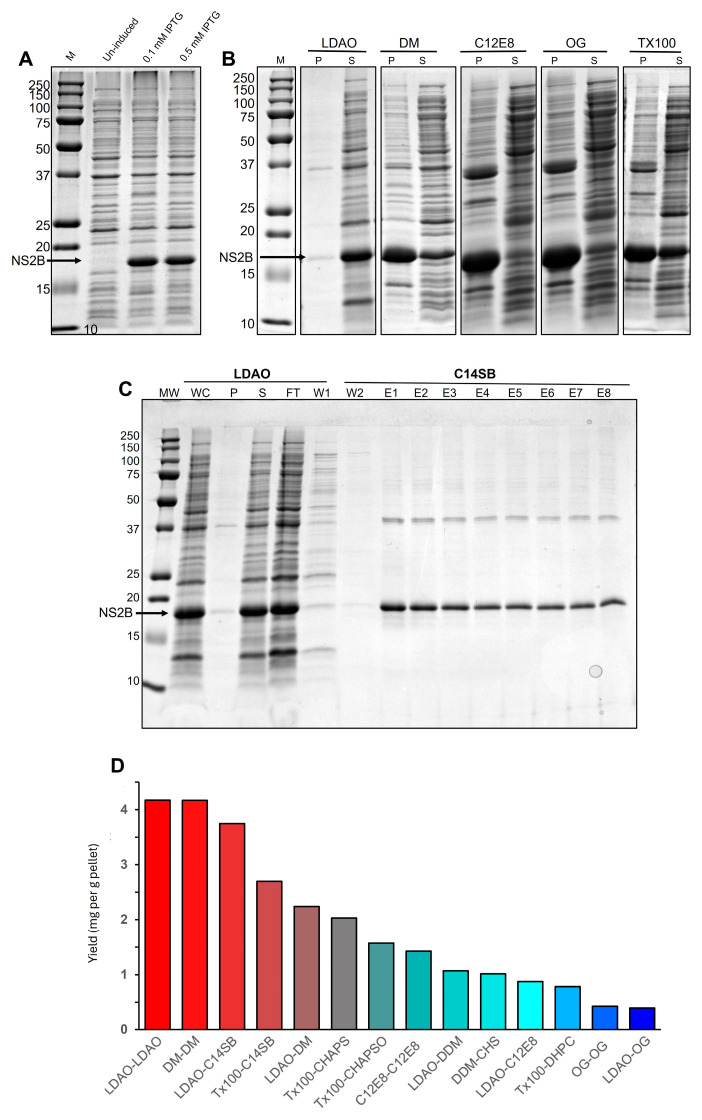
**Purification of NS2B protein.** (**A**) SDS-PAGE before and after IPTG induction; (**B**) stitched gels assessing extraction efficiency in pellet (P) and supernatant (S) samples after sonication with detergent and centrifugation; (**C**) example of purification using Ni-NTA in the system LDAO (extraction)-C14SB (purification), with lanes corresponding to whole cell lysate (WC), flow-through (FT), pellet (P), supernatant (S), flowthrough (FT), wash 1 (W1), wash buffer + C14SB (W2) and elution fractions in C14SB (E1–E8); (**D**) yield of NS2B for different combinations of detergents used for extraction and Ni-NTA purification: e.g., ‘LDAO-C14SB’ means extraction in LDAO and purification in C14SB.

After IPTG addition, a thick band at ~17 kDa was observed consistent with the NS2B monomer (Figure 2A). Of the detergents tested, LDAO was the best detergent for extraction, since minimal NS2B was present in pellet fractions after solubilization in this detergent (Figure 2B). After Ni-NTA resin purification, the elution samples were reasonably pure after LDAO extraction and C14SB elution (Figure 2C). The best combination for extraction and purification in Ni-NTA resin was LDAO-LDAO, which produced a yield of >4 mg NS2B per gram of cell pellet (Figure 2D). Since we obtained ~4.5 g of pellet for a 0.5 L culture, this results in a yield of ~36 mg of NS2B/L of culture. A similar high yield was achieved for detergent pairs DM-DM or LDAO-C14SB (pairs refer to those used in extraction and purification steps, respectively) (Figure 2C). Some earlier ZIKV NS2B purifications did not report any yield in LMPG [25] or SDS [45], although DENV NS2B was obtained at 3 mg/L of culture in LMPG [46] and 15 mg/L of culture after purifying from inclusion bodies using urea and SDS before refolding in LMPG micelles.

### 2.2. Purification by Size Exclusion Chromatography (SEC)

**Figure 3:**
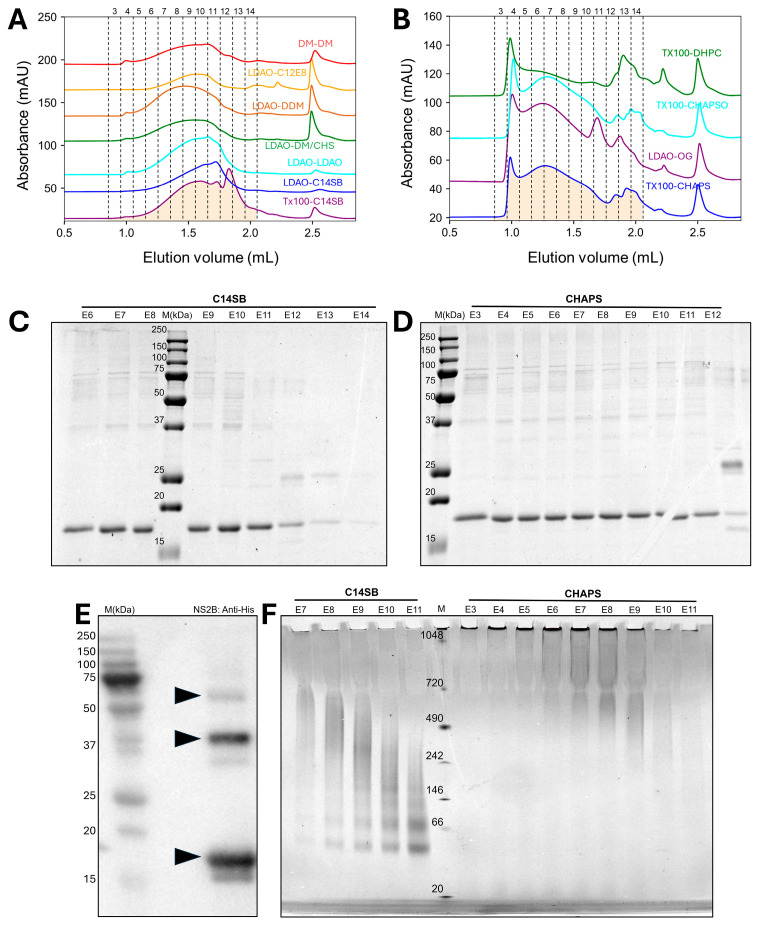
**Oligomerization of NS2B in SEC.** (**A**,**B**) SEC profiles in the detergents indicated (used in extraction and small-scale purification in a Superose 6 column (3.2/300), as in Figure 2D), grouped into those producing high MW species (**A**) and low MW ones (**B**); (**C**,**D**) SDS gels of SEC fractions from C14SB (**C**) and CHAPS purification (**D**); (**E**) Western blot of NS2B SEC fraction 3 from C12E8-C12E8 SEC; (**F**) Blue native PAGE of SEC fractions from C14SB and CHAPS purification.

When NS2B obtained from Ni-NTA purification was further purified via SEC, elution profiles were very broad in all detergent combinations tested, suggesting sample heterogeneity in the oligomers present. In some of the conditions, the protein eluted late (~1.5 to 2 mL) (Figure 3A), likely representing smaller oligomers. In other detergents, most elution appeared at or near the void volume (~1 to 1.5 mL, Figure 3B), suggesting larger aggregates. Regardless of the type of profile, e.g., late (C14SB) or early (CHAPS) elution, these fractions produced mostly monomers in SDS-PAGE (Figure 3C,D), although some possible dimers and trimers were also present (~34 kDa and 51 kDa, respectively). The latter was confirmed in a Western blot (Figure 3E). However, a non-denaturing BN-PAGE showed a clear difference between late and early elution types of profile (Figure 3F): in C14SB, one can detect from monomers up to tetramers, whereas in CHAPS there were smears consistent with much larger oligomers (500–1000 kDa), and some protein was even unable to enter the gel, representing very large aggregates.

### 2.3. Crosslinking Results

**Figure 4:**
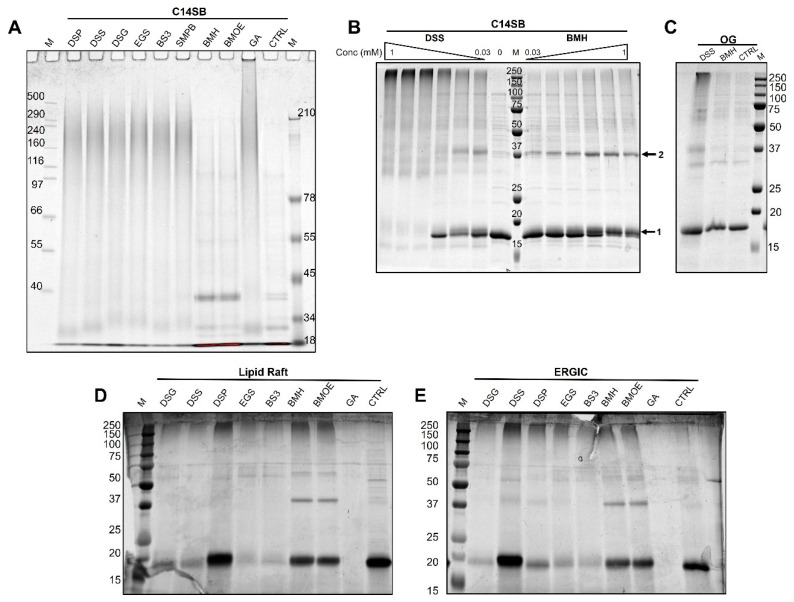
**Crosslinking of NS2B protein.** (**A**) Screening of crosslinkers at 1 mM in 7% Tris-acetate gel in C14SB; (**B**) crosslinking by serial dilutions of DSS and BMH; (**C**) crosslinking with 0.03 mM BMH and DSS in OG; (**D**,**E**) screening of 1 mM crosslinkers in lipid rafts (**D**) and ERGIC (**E**).

To test if NS2B could be crosslinked in C14SB detergent, we tested amine crosslinkers (DSG, DSS, DSP, EGS, BS3, GA), SMBP (a bifunctional amine-cysteine crosslinker) and cysteine crosslinkers BMH and BMOE. While most crosslinkers caused high MW smears with no discernible bands (Figure 4A), BMH and BMOE produced ladder-like patterns. Two of these detergents (DSS and BMH) were titrated from 0.03 to 1 mM (Figure 4B). Without crosslinker, the sample produced mostly monomers, but increasing concentration of DSS produced from dimers to very large oligomers. In contrast, BMH produced mostly monomers and dimers. In other detergents, e.g. OG, crosslinking with DSS and BMH produced similar results (Figure 4C). When crosslinking was performed in a ‘lipid raft mixture’ (Figure 4D), some crosslinkers produced almost no monomer (e.g., DSG, DSS, EGS, BS3 and GA) suggesting very large species unable to enter the gel. Similar results were obtained in ERGIC mixture (Figure 4E). Since we observed a gel migration consistent with large, crosslinked oligomers in both detergent and liposomes, we then tested oligomerization in detergent without crosslinking, using gold-standard biophysical techniques mass photometry and analytical ultracentrifugation.

### 2.4. Mass Spectrometry Results

**Figure 5:**
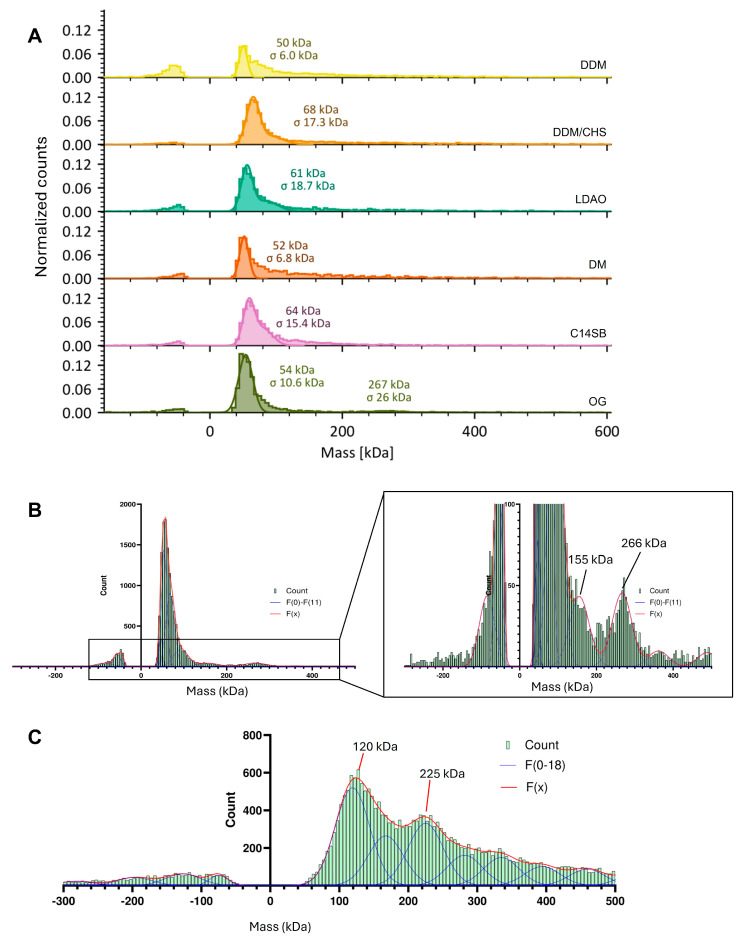
**Mass photometry.** (**A**) Mass photometry results for NS2B in LMNG after preincubation in the detergents indicated; (**B**) sum of multiple profiles in independent samples after OG preincubation; (**C**) result after OG preincubation using a Refeyn Microfluidics Chip.

Samples of NS2B protein in different detergents were then reconstituted in low cmc detergent LMNG, which largely remains attached to the protein even after dilution below cmc during the short period required for MP measurements. In all cases, a large peak at ~50–70 kDa was observed (Figure 5A), followed by a featureless distribution at higher MW. However, when we added the measurements of five independent samples prepared on different days in OG, we noticed at least two obvious bands arising from the noise, at approximately 155 and 266 kDa (Figure 5B), and even larger broadly distributed species. We note that the reported mass of these particles also includes the bound LMNG detergent. A similar addition of various independent profiles performed for other detergents did not result in a discernible discrete species. We then tested these samples using a Refeyn microfluidics chip accessory, where the protein can be tested in OG at a higher initial concentration, and where detergent is completely removed. In this case, similar peaks were observed at 120 kDa and 225 kDa, consistent with the same peaks described above, but as indicated, without detergent contribution (Figure 5C). Comparison of these MW with the ones in LMNG, the latter contributes about ~35 kDa to the particle MW. Since the NS2B monomer is ~17 kDa, we speculate that 120 and 225 kDa correspond to 6-mers and 12-mers, respectively.

### 2.5. Analytical Ultracentrifugation

**Figure 6:**
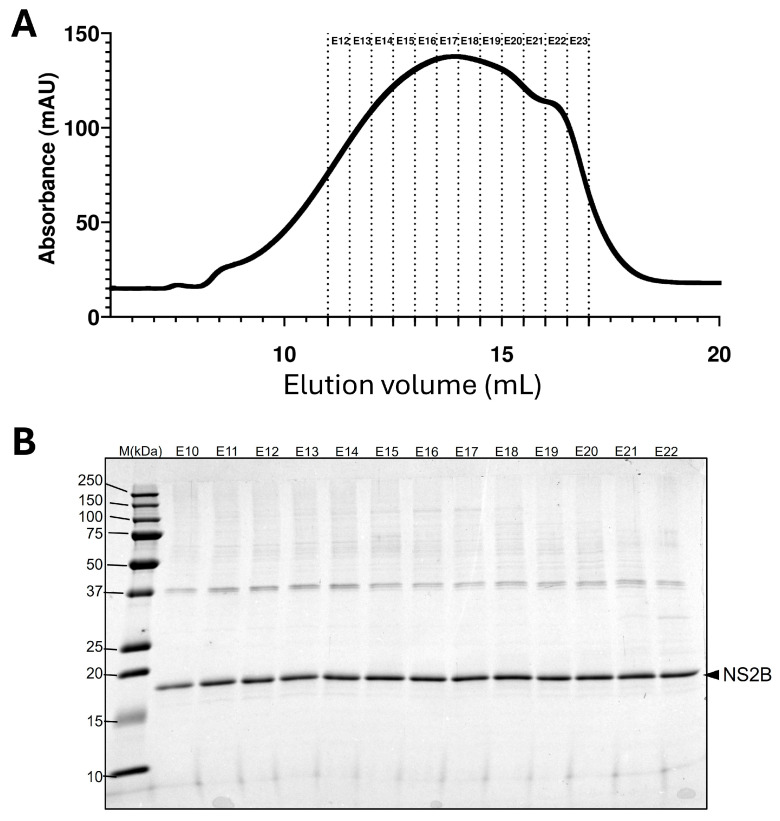
**Comparison of early and late fractions in SEC.** (**A**) SEC profile and fractions E13–E22 used for AUC SV obtained in a Superose 6 column (10/300); (**B**) Coomassie-stained gel corresponding to fractions E10–E22 showing successful NS2B purification.

**Figure 7:**
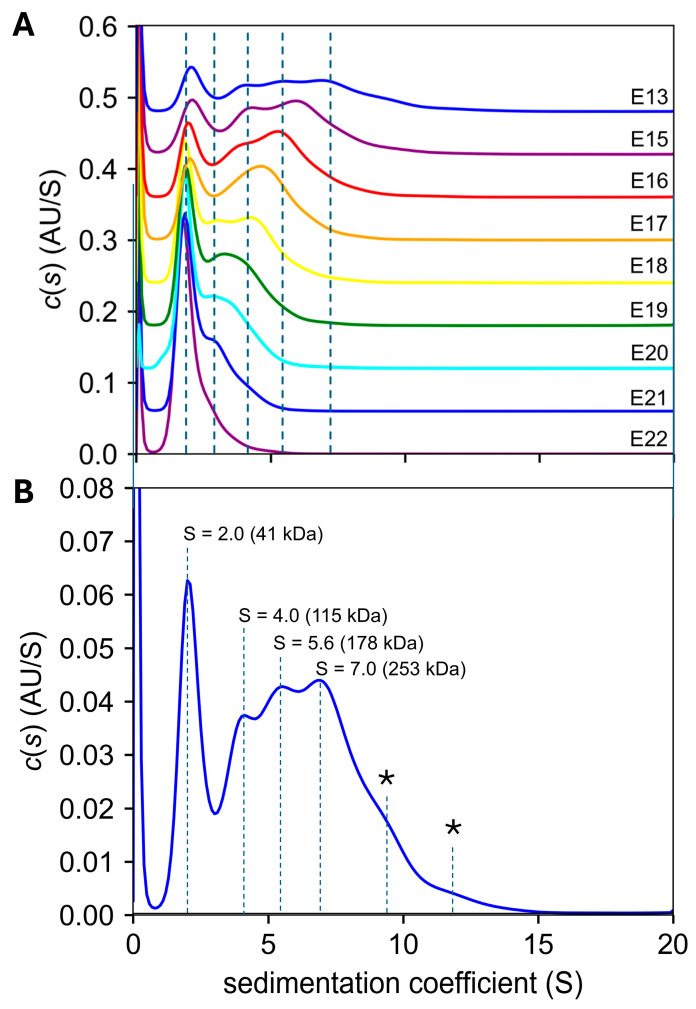
**AUC SV profiles from SEC fractions.** (**A**) SV profiles of the fractions shown in Figure 6 from E13 (earlier fraction) to E22 (later fraction), shown staggered for clarity. The dotted lines correspond to the main bands observed for E13; (**B**) detail of fraction E13, with indicated S values and predicted molecular weights for the main four bands, although another two bands are observed with even higher S values (*), at approximately S = 9.5 (412 kDa) and S = 12 (580 kDa).

AUC-SV experiments were conducted in C14SB detergent, using all the LDAO-C14SB SEC fractions (Figure 6A) that showed no contaminant bands (Figure 6B). We estimated that the detergent-to-protein (DPR) molar ratio in these fractions was 125–170. In all fractions, the c(s) distribution showed a band around S = 2 (Figure 7A) which corresponds to a particle of ~40 kDa, consistent with dimers. In the latest fraction, E22, this was the only species present. Fractions eluting earlier (up to E13) showed species with progressively larger S values. The c(s) profile for the earliest eluting fraction (Figure 7B) shows increasing S values consistent with multiples of the 17.3 kDa monomer (2.36, 6.64, 10.2 and 14.6) possibly corresponding to dimers, hexamers, decamers and tetradecamers.

Electron microscopy negative staining images of NS2B in C14SB proved unsuccessful, producing amorphous shapes.

## 3. Discussion

Our detergent screening shows that the best detergents for ZIKV NS2B purification are small zwitterionic detergents, like LDAO and C14SB, where the protein tends to form smaller oligomers, as shown in SEC data and BN-PAGE.

Crosslinking in detergent was an extension of a previous study that only used GA in LMPG micelles [25]. Here, we used many other crosslinkers of different reactivity and solubility. We found that maleimide-based crosslinkers were less effective than *N*-Hydroxy succinimide (NHS) ester-based crosslinkers. In liposomes, crosslinking was so extensive that the protein was unable to enter the gel in some cases. Mass photometry in detergent always produced a broad featureless range of molecular weight distributions regardless of the detergent used, but nevertheless indicative of large multimers. However, in OG, clear discrete bands could be observed corresponding roughly to hexamers and dodecamers. Similar results were observed when using a microfluidics system without the presence of detergent. Lastly, AUC-SV experiments again showed that NS2B exists in a very broad range of oligomeric states in C14SB detergent, represented in SEC by a very broad elution profile. The fact that these oligomers were also observed in AUC shows that, once formed, they are relatively stable. These oligomers are likely stabilized by a network of interactions between NS2B α-helical hairpins [25,31], a motif also found in reticulons [47]. The latter have ‘reticulon homology domains (RHDs)’ that can modulate the shape of ER membranes, contributing to the formation of tubules via formation of a wedge in the cytoplasmic side of the ER membrane. For example, reticulon Rtn4a has been demonstrated to self-oligomerize, as shown by crosslinking, density-gradient centrifugation and immunofluorescence experiments [48], whereas their overexpression reduced the diameter of ER tubules. Overexpression of RHD-containing FAM134B induced ER fragmentation [49]. Since RHD-containing proteins require oligomerization to produce these effects, we hypothesize that NS2B may exert an ER-remodeling effect via a similar mechanism.

Unfortunately, the structure of these NS2B oligomers is unknown, and AF2 or AF3 could not obtain reliable models, even for dimers. We have shown previously for ZIKV NS4B [50] that AF could correctly predict the length and position of the nine α-helical segments reported experimentally by solution NMR [7,51], therefore it is likely that the secondary structure prediction for the NS2B monomeric form is largely correct. However, the overall 3D conformation of the monomer is uncertain. Indeed, in the paper referred to above [50], AF predicted short hydrophobic domains or a very tilted structure, whereas the experimental model consists of regular α-helices. Similarly, in the case of ZIKV NS4A [52], AF predicted a regular three-helix bundle for the monomer, whereas, in the experimental model, the second α-helix does not span the membrane [19,53]. Therefore, we conclude that the inability of finding reliable oligomeric models is caused by incorrect prediction of monomer folding.

Interestingly, the NS2B-NS3 protease complex can cleave the host FAM134B, inhibiting ER remodeling and reticulophagy [54], whereas silencing of FAM134B directly led to increased ZIKV viral titers in cell infection assays, pointing to a possible virus-induced cellular control to enhance replication. However, the precise mechanism for FAM134B recognition and cleavage by NS2B-NS3 is not known, and it remains unknown if the NS2B hydrophobic domains play any role in this mechanism.

Other candidate binding partners of NS2B include the lunapark family of proteins that stabilize network junctions in the ER [55] or atlastins which promote ER fusion, antagonizing the ER-fragmenting effects of reticulons [56]. In fact, interplay between reticulons, atlastins, and lunapark proteins is key in guiding overall ER shape and structure [57,58]. Hence, NS2B protein, via its extreme oligomerization tendency, could modulate the ER by perturbing the subtle balance between these three protein families.

## 4. Materials and Methods

### 4.1. Protein Purification

Plasmid containing the full-length sequence of ZIKV NS2B with an N-terminal S-tag and thrombin cleavage sequence and a C-terminal 6xHistidine tag (generously shared by Dr Congbao Kang [25]) were transformed into competent BL21-Codon Plus (DE3)-RIPL *E. coli* by heat shock at 42 °C for 20 s. Transformed cells were plated on lysogeny broth (LB) agar with kanamycin and chloramphenicol and were grown overnight. Single colonies were picked and inoculated in 50 mL Terrific Broth (TB) complex media [59] at 37 °C overnight. Starter culture (5 mL) was added to 500 mL of TB and grown at 37 °C. Cultures were induced with IPTG at ~1.6 OD, incubated for 16 h at 18 °C and centrifuged at 7500× *g* for 10 min at 4 °C. The resulting pellet was collected and frozen in liquid nitrogen at −80 °C. *E. coli* pellets were resuspended in lysis buffer (1 mL buffer per 0.1 g pellet) supplemented with benzonase nuclease, lysozyme and protease inhibitor. The solution was sonicated in an ice bath for 10 min. Detergent was added before sonication on ice for another 10 min. Sonicated samples were centrifuged at 40,000× *g* for 30 min at 4 °C. The supernatant was incubated with Ni-NTA resin for 2 h at 4 °C and was collected with a spin column. The resin was washed, and bound protein was eluted. Protein concentrations were measured using a NanoDrop One UV-Vis Spectrophotometer (ThermoFisher Scientific, Singapore). Ni-NTA elutions were concentrated with an Amicon Ultra 10 kDa cutoff concentrator (Merck Millipore, Burlington, MA, USA) and further purified using size exclusion chromatography (SEC) using a Superdex 6 Increase column 3.2/300 or 10/300 (Cytiva, Marlborough, MA, USA) on an AKTA system (Cytiva, Marlborough, MA, USA) with buffer containing 20 mM sodium phosphate pH 7.1, 150 mM KCl, and 1 mM Tris(2-carboxyethyl)phosphine (TCEP). Detergents were added to the SEC buffer.

### 4.2. Electrophoresis

Sodium dodecyl sulfate–polyacrylamide gel electrophoresis (SDS-PAGE) and blue native PAGE was performed as reported previously [60,61]. For Tris-acetate GE, samples were loaded on a 7% Tris-acetate precast gel with Tris-tricine-SDS gel running buffer (50 mM Tris, 50 mM tricine, 0.1% SDS) and run at 150 V for 1 h. Tris-acetate gels were stained and destained according to the reported SDS-PAGE protocol. Western blotting was performed using a Trans-Blot^®^ SD Semi-Dry Transfer Cell (BioRad, Hercules, CA, USA) onto a PVDF membrane (BioRad, USA). Detection was performed using rabbit polyclonal anti-His primary antibody and HRP-conjugated goat anti-rabbit secondary antibody. Bands were visualized using Pierce ECL Western blotting substrate (ThermoFisher Scientific, Singapore). All gels and blots were imaged on an Invitrogen iBright FL1500 Imaging System (ThermoFisher Scientific, Singapore).

### 4.3. Crosslinking in Detergents and Liposomes

NS2B (3.5 μg in 9.5 μL) in detergent was mixed with 0.5 μL of crosslinker (either DSP, DSS, DSG, EGS, BS3, SMBP, BMH, BMOE or GA) at various concentrations (from ~0.6 to 20 mM). Samples were incubated for 10 min before quenching with 2 μL of 0.5 M Tris pH 8.0, except samples containing BMH and BMOE which were quenched with 0.5 M DTT. Subsequently, 4 μL of 4× LDS sample buffer or 3 μL 5× TGS sample buffer (250 mM Tris pH 6.8, 50% glycerol, 10% SDS, 0.5% Bromophenol Blue, 500 mM DTT) was added to the samples before heating at 70 °C for 10 min. LDS and TGS samples were run in Tris-acetate gels and Tris-glycine gels, respectively, with various polyacrylamide concentrations. For crosslinking in liposomes, NS2B in detergent was first mixed with either ERGIC mixture (POPC, POPE, Chol, liver PI, POPS at a molar ratio 45:20:15:13:7) or a ’lipid raft mixture’ (DOPC, Sphyn, Chol at a molar ratio 40:40:20), at a 25:1 lipid-to-protein molar ratio. Before crosslinking, the detergent was removed from the solution using BioBeads (BioRad, Hercules, CA, USA).

### 4.4. Mass Photometry

Mass photometry was performed using a Refeyn TwoMP Mass Photometer (Refeyn, Oxford, UK), operated according to manufacturer guidelines. For manual measurements, 2 µL of a ‘10× solution’ containing 250 nM NS2B in SEC buffer and 20 μM LMNG was diluted (10×) in 18 μL SEC buffer without detergent to produce 20 µL of 25 nM NS2B in 2 μM LMNG. Samples were measured immediately after dilution. Measurements using the Mass Fluidix HC system were performed according to manufacturer guidelines. NS2B (38 μM) in SEC buffer with 9% OG was rapidly diluted (2000×) with filtered SEC buffer containing no detergent (to 19 nM) using a microfluidics system before measurements were recorded. Data analysis was performed using the AcquireMP (v. 2025 R1) and DiscoverMP software (v. 2025 R1) (Refeyn, Oxford, UK) and Fityk curvefitting software (v. 1.3.2) [62] and visualized using GraphPad Prism (v. 10.1.2).

### 4.5. Analytical Ultracentrifugation (AUC)

Sedimentation velocity experiments were performed in a Beckman Coulter ProteomeLab XL-I analytical ultracentrifuge (Brea, CA, USA) with a 50-Ti rotor. Given the heterogeneity of the SEC profiles, we used each of the SEC fractions obtained in 0.1% C14SB. The detergent contribution was density-matched with D_2_O (33.4%) [52,63]. Samples were centrifuged from 40,000 to 48,000 rpm and scans were collected at 280 nm at 5 min intervals for 10 h. The data was analyzed using SEDFIT (v. 18.1) [64] and visualized with GUSSI (v. 1.0.8d) [65]. Buffer densities and viscosities were calculated using SEDNTERP (v. 3) [66].

### 4.6. Structure Prediction

Structure prediction of ZIKV NS2B was obtained from an AF3 server or a ColabFold (AF2) notebook, producing essentially the same result. AF3 prediction was performed using the AlphaFold Server (https://alphafoldserver.com) [67]. After the process was finished, we selected the top-ranked model. The PAE Viewer web server was used to generate and evaluate PAE plots [68]. When using the ColabFold notebook (AF2), we used ColabFold (ColabFold v1.5.5: AlphaFold2 [69,70]) as described [71]. We used the default MMseqs2 multiple sequence alignment (MSA), which produced MSAs with >100 sequences, sufficient for a reliable prediction (<30) [70]. The best model in each run was energy minimized by OpenMM/Amber (relax_amber.ipynb), using default values of 2000 max_iterations, tolerance 2.39 and stiffness 10 [69]. Predictions for NS2B oligomers (from dimers to dodecamers) did not produce any reliable structure. Graphical representation and visualization were performed in Chimera X [72,73]. The structure of the NS2B monomer was also predicted using the Robetta tool in RoseTTAFold (https://robetta.bakerlab.org).

## 5. Conclusions

We have investigated the extraction, purification and oligomeric behavior of ZIKV NS2B in detergent environments and in liposomes.

Crosslinking, mass photometry and AUC-SV reveal that very large oligomers are formed by NS2B.

We speculate that the structural similarities to reticulons and other ER-modifying proteins may define a similar role for NS2B in ER remodeling that promotes viral replication.

## Data Availability

The datasets generated during and/or analyzed during the current study are available from the corresponding author on reasonable request.

## References

[1] Stevens A.J., Gahan M.E., Mahalingam S., Keller P.A. (2009). The Medicinal Chemistry of Dengue Fever. J. Med. Chem..

[2] Pielnaa P., Al-Saadawe M., Saro A., Dama M.F., Zhou M., Huang Y., Huang J., Xia Z. (2020). Zika virus-spread, epidemiology, genome, transmission cycle, clinical manifestation, associated challenges, vaccine and antiviral drug development. Virology.

[3] Ribeiro G.S., Kitron U. (2016). Zika virus pandemic: A human and public health crisis. Rev. Soc. Bras. Med. Trop..

[4] Miller S., Sparacio S., Bartenschlager R. (2006). Subcellular localization and membrane topology of the Dengue virus type 2 Non-structural protein 4B. J. Biol. Chem..

[5] Nemesio H., Palomares-Jerez F., Villalain J. (2012). NS4A and NS4B proteins from dengue virus: Membranotropic regions. Biochim. Biophys. Acta.

[6] Zou J., Xie X., Lee L.T., Chandrasekaran R., Reynaud A., Yap L., Wang Q.Y., Dong H., Kang C., Yuan Z. (2014). Dimerization of flavivirus NS4B protein. J. Virol..

[7] Li Y., Wong Y.L., Lee M.Y., Li Q., Wang Q.Y., Lescar J., Shi P.Y., Kang C. (2016). Secondary structure and membrane topology of the full-length dengue virus NS4B in micelles. Angew. Chem..

[8] Porter S.S., Gilchrist T.M., Schrodel S., Tai A.W. (2025). Dengue and Zika virus NS4B proteins differ in topology and in determinants of ER membrane protein complex dependency. J. Virol..

[9] Kuhn R.J., Zhang W., Rossmann M.G., Pletnev S.V., Corver J., Lenches E., Jones C.T., Mukhopadhyay S., Chipman P.R., Strauss E.G. (2002). Structure of dengue virus: Implications for flavivirus organization, maturation, and fusion. Cell.

[10] Mukhopadhyay S., Kim B.S., Chipman P.R., Rossmann M.G., Kuhn R.J. (2003). Structure of West Nile virus. Science.

[11] Zhang Y., Kaufmann B., Chipman P.R., Kuhn R.J., Rossmann M.G. (2007). Structure of immature West Nile virus. J. Virol..

[12] Xie X., Zou J., Zhang X., Zhou Y., Routh A.L., Kang C., Popov V.L., Chen X., Wang Q.Y., Dong H. (2019). Dengue NS2A Protein Orchestrates Virus Assembly. Cell Host Microbe.

[13] Klema V.J., Padmanabhan R., Choi K.H. (2015). Flaviviral replication complex: Coordination between RNA synthesis and 5′-RNA capping. Viruses.

[14] van den Elsen K., Chew B.L.A., Ho J.S., Luo D. (2023). Flavivirus nonstructural proteins and replication complexes as antiviral drug targets. Curr. Opin. Virol..

[15] Welsch S., Miller S., Romero-Brey I., Merz A., Bleck C.K.E., Walther P., Fuller S.D., Antony C., Krijnse-Locker J., Bartenschlager R. (2009). Composition and Three-Dimensional Architecture of the Dengue Virus Replication and Assembly Sites. Cell Host Microbe.

[16] Miorin L., Romero-Brey I., Maiuri P., Hoppe S., Krijnse-Locker J., Bartenschlager R., Marcello A. (2013). Three-dimensional architecture of tick-borne encephalitis virus replication sites and trafficking of the replicated RNA. J. Virol..

[17] Romero-Brey I., Bartenschlager R. (2016). Endoplasmic Reticulum: The Favorite Intracellular Niche for Viral Replication and Assembly. Viruses.

[18] Miller S., Krijnse-Locker J. (2008). Modification of intracellular membrane structures for virus replication. Nat. Rev. Microbiol..

[19] Miller S., Kastner S., Krijnse-Locker J., Bühler S., Bartenschlager R. (2007). The non-structural protein 4A of dengue virus is an integral membrane protein inducing membrane alterations in a 2K-regulated manner. J. Biol. Chem..

[20] Kuno G., Chang G.J.J. (2007). Full-length sequencing and genomic characterization of Bagaza, Kedougou, and Zika viruses. Arch. Virol..

[21] Lindenbach B.D., Thiel H.J., Rice C.M. (2007). Flaviviridae: The viruses and their replication. Fields Virology.

[22] Gould E., Solomon T. (2008). Pathogenic flaviviruses. Lancet.

[23] Kuno G., Chang G.J.J., Tsuchiya K.R., Karabatsos N., Cropp C.B. (1998). Phylogeny of the genus Flavivirus. J. Virol..

[24] Pierson T.C., Diamond M.S. (2020). The continued threat of emerging flaviviruses. Nat. Microbiol..

[25] Ng E.Y., Loh Y.R., Li Y., Li Q., Kang C. (2019). Expression, purification of Zika virus membrane protein-NS2B in detergent micelles for NMR studies. Protein Expr. Purif..

[26] Penna B.R., Gomes-Neto F., Anobom C.D., Valente A.P. (2024). Structural and dynamics characterization of the Zika virus NS2B using nuclear magnetic resonance and RosettaMP: A challenge for transmembrane protein studies. Int. J. Biol. Macromol..

[27] Krogh A., Larsson B., von Heijne G., Sonnhammer E.L. (2001). Predicting transmembrane protein topology with a hidden Markov model: Application to complete genomes. J. Mol. Biol..

[28] Sonnhammer E.L., von Heijne G., Krogh A. (1998). A hidden Markov model for predicting transmembrane helices in protein sequences. Proc. Int. Conf. Intell. Syst. Mol. Biol..

[29] Li X.D., Deng C.L., Ye H.Q., Zhang H.L., Zhang Q.Y., Chen D.D., Zhang P.T., Shi P.Y., Yuan Z.M., Zhang B. (2016). Transmembrane domains of NS2B contribute to both viral RNA replication and particle formation in Japanese encephalitis virus. J. Virol..

[30] Huang Q., Li Q., Joy J., Chen A.S., Ruiz-Carrillo D., Hill J., Lescar J., Kang C. (2013). Lyso-myristoyl phosphatidylcholine micelles sustain the activity of Dengue non-structural (NS) protein 3 protease domain fused with the full-length NS2B. Protein Expr. Purif..

[31] Li Y., Li Q., Wong Y.L., Liew L.S.Y., Kang C. (2015). Membrane topology of NS2B of dengue virus revealed by NMR spectroscopy. Biochim. Biophys. Acta Biomembr..

[32] Kiemel D., Kroell A.-S.H., Denolly S., Haselmann U., Bonfanti J.-F., Andres J.I., Ghosh B., Geluykens P., Kaptein S.J.F., Wilken L. (2024). Pan-serotype dengue virus inhibitor JNJ-A07 targets NS4A-2K-NS4B interaction with NS2B/NS3 and blocks replication organelle formation. Nat. Commun..

[33] De La Cruz L., Nguyen T.H.D., Ozawa K., Shin J., Graham B., Huber T., Otting G. (2011). Binding of low molecular weight inhibitors promotes large conformational changes in the dengue virus ns2b-ns3 protease: Fold analysis by pseudocontact shifts. J. Am. Chem. Soc..

[34] Noble C.G., Seh C.C., Chao A.T., Shi P.Y. (2012). Ligand-bound structures of the dengue virus protease reveal the active conformation. J. Virol..

[35] Kim Y.M., Gayen S., Kang C., Joy J., Huang Q., Chen A.S., Wee J.L., Ang M.J., Lim H.A., Hung A.W. (2013). NMR analysis of a novel enzymatically active unlinked dengue NS2B-NS3 protease complex. J. Biol. Chem..

[36] Li Y., Loh Y.R., Hung A.W., Kang C. (2018). Characterization of molecular interactions between Zika virus protease and peptides derived from the C-terminus of NS2B. Biochem. Biophys. Res. Commun..

[37] Ni X., Richardson R.B., Godoy A.S., Ferla M.P., Kikawa C., Scheen J., Hannon W.W., Capkin E., Lahav N., Balcomb B.H. (2025). Combined crystallographic fragment screening and deep mutational scanning enable discovery of Zika virus NS2B-NS3 protease inhibitors. Nat. Commun..

[38] Phoo W.W., Li Y., Zhang Z., Lee M.Y., Loh Y.R., Tan Y.B., Ng E.Y., Lescar J., Kang C., Luo D. (2016). Structure of the NS2B-NS3 protease from Zika virus after self-cleavage. Nat. Commun..

[39] Li X.D., Li X.F., Ye H.Q., Deng C.L., Ye Q., Shan C., Shang B.D., Xu L.L., Li S.H., Cao S.B. (2014). Recovery of a chemically synthesized Japanese encephalitis virus reveals two critical adaptive mutations in NS2B and NS4A. J. Gen. Virol..

[40] Choksupmanee O., Hodge K., Katzenmeier G., Chimnaronk S. (2012). Structural platform for the autolytic activity of an intact NS2B-NS3 protease complex from dengue virus. Biochemistry.

[41] Zou J., Lee L.T., Wang Q.Y., Xie X., Lu S., Yau Y.H., Yuan Z., Geifman Shochat S., Kang C., Lescar J. (2015). Mapping the Interactions between the NS4B and NS3 proteins of dengue virus. J. Virol..

[42] Zou J., Xie X., Wang Q.Y., Dong H., Lee M.Y., Kang C., Yuan Z., Shi P.Y. (2015). Characterization of dengue virus NS4A and NS4B protein interaction. J. Virol..

[43] Surya W., Goh J., Ponniah C., Torres J. (2025). AlphaFold Prediction of Protein-Protein Interactions in the Flaviviridae Proteomes. Int. J. Mol. Sci..

[44] Uchil P.D., Satchidanandam V. (2003). Architecture of the flaviviral replication complex: Protease, nuclease, and detergents reveal encasement within double-layered membrane compartments. J. Biol. Chem..

[45] Penna B.R., de Oliveira D.M.P., Anobom C.D., Valente A.P. (2022). Backbone 1H, 15N, and 13C resonance assignments of the non-structural protein NS2B of Zika virus. Biomol. NMR Assign..

[46] Huang Q., Chen A.S., Li Q., Kang C. (2011). Expression, purification, and initial structural characterization of nonstructural protein 2B, an integral membrane protein of Dengue-2 virus, in detergent micelles. Protein Expr. Purif..

[47] Yang Y.S., Strittmatter S.M. (2007). The reticulons: A family of proteins with diverse functions. Genome Biol..

[48] Zurek N., Sparks L., Voeltz G. (2011). Reticulon Short Hairpin Transmembrane Domains Are Used to Shape ER Tubules. Traffic.

[49] Bhaskara R.M., Grumati P., Garcia-Pardo J., Kalayil S., Covarrubias-Pinto A., Chen W., Kudryashev M., Dikic I., Hummer G. (2019). Curvature induction and membrane remodeling by FAM134B reticulon homology domain assist selective ER-phagy. Nat. Commun..

[50] Surya W., Tan P., Honey S.S., Mehta D., Torres J. (2025). Flavivirus NS4B proteins do not form homodimers: Discrepancies with an AlphaFold-based oligomeric model. Comput. Struct. Biotechnol. J..

[51] Li Y., Kim Y.M., Zou J., Wang Q.Y., Gayen S., Wong Y.L., Lee L.T., Xie X., Huang Q., Lescar J. (2015). Secondary structure and membrane topology of dengue virus NS4B N-terminal 125 amino acids. Biochim. Biophys. Acta–Biomembr..

[52] Surya W., Honey S.S., Torres J. (2024). Flavivirus Zika NS4A protein forms large oligomers in liposomes and in mild detergent. Sci. Rep..

[53] Li Y., Lee M.Y., Loh Y.R., Kang C. (2018). Secondary structure and membrane topology of dengue virus NS4A protein in micelles. Biochim. Biophys. Acta—Biomembr..

[54] Lennemann N.J., Coyne C.B. (2017). Dengue and Zika viruses subvert reticulophagy by NS2B3-mediated cleavage of FAM134B. Autophagy.

[55] Wang S., Powers R.E., Gold V.A., Rapoport T.A. (2018). The ER morphology-regulating lunapark protein induces the formation of stacked bilayer discs. Life Sci. Alliance.

[56] Hu X., Wu F., Sun S., Yu W., Hu J. (2015). Human atlastin GTPases mediate differentiated fusion of endoplasmic reticulum membranes. Protein Cell.

[57] Espadas J., Pendin D., Bocanegra R., Escalada A., Misticoni G., Trevisan T., Velasco Del Olmo A., Montagna A., Bova S., Ibarra B. (2019). Dynamic constriction and fission of endoplasmic reticulum membranes by reticulon. Nat. Commun..

[58] Wang S., Tukachinsky H., Romano F.B., Rapoport T.A. (2016). Cooperation of the ER-shaping proteins atlastin, lunapark, and reticulons to generate a tubular membrane network. eLife.

[59] Studier F.W. (2005). Protein production by auto-induction in high-density shaking cultures. Protein Expr. Purif..

[60] Gan S.W., Vararattanavech A., Nordin N., Eshaghi S., Torres J. (2011). A cost-effective method for simultaneous homo-oligomeric size determination and monodispersity conditions for membrane proteins. Anal. Biochem..

[61] To J., Torres J. (2021). Trimerisation of the N-terminal tail of Zika virus NS4A protein: A potential in vitro antiviral screening assay. Membranes.

[62] Wojdyr M. (2010). Fityk: A general-purpose peak fitting program. J. Appl. Crystallogr..

[63] Surya W., Torres J. (2015). Sedimentation equilibrium of a small oligomer-forming membrane protein: Effect of histidine protonation on pentameric stability. J. Vis. Exp. JoVE.

[64] Schuck P., Perugini M.A., Gonzales N.R., Howlett G.J., Schubert D. (2002). Size-distribution analysis of proteins by analytical ultracentrifugation: Strategies and application to model systems. Biophys. J..

[65] Brautigam C.A. (2015). Calculations and Publication-Quality Illustrations for Analytical Ultracentrifugation Data. Methods Enzymol..

[66] Philo J.S. (2023). SEDNTERP: A calculation and database utility to aid interpretation of analytical ultracentrifugation and light scattering data. Eur. Biophys. J..

[67] Abramson J., Adler J., Dunger J., Evans R., Green T., Pritzel A., Ronneberger O., Willmore L., Ballard A.J., Bambrick J. (2024). Accurate structure prediction of biomolecular interactions with AlphaFold 3. Nature.

[68] Elfmann C., Stülke J. (2023). PAE viewer: A webserver for the interactive visualization of the predicted aligned error for multimer structure predictions and crosslinks. Nucleic Acids Res..

[69] Mirdita M., Schutze K., Moriwaki Y., Heo L., Ovchinnikov S., Steinegger M. (2022). ColabFold: Making protein folding accessible to all. Nat. Methods.

[70] Jumper J., Evans R., Pritzel A., Green T., Figurnov M., Ronneberger O., Tunyasuvunakool K., Bates R., Žídek A., Potapenko A. (2021). Highly accurate protein structure prediction with AlphaFold. Nature.

[71] Torres J., Pervushin K., Surya W. (2024). Prediction of conformational states in a coronavirus channel using Alphafold-2 and DeepMSA2: Strengths and limitations. Comput. Struct. Biotechnol. J..

[72] Pettersen E.F., Goddard T.D., Huang C.C., Couch G.S., Greenblatt D.M., Meng E.C., Ferrin T.E. (2004). UCSF Chimera—A visualization system for exploratory research and analysis. J. Comput. Chem..

[73] Pettersen E.F., Goddard T.D., Huang C.C., Meng E.C., Couch G.S., Croll T.I., Morris J.H., Ferrin T.E. (2021). UCSF ChimeraX: Structure visualization for researchers, educators, and developers. Protein Sci..

